# Experiences with Take it Personal!+ from People with Mild Intellectual Disability or Borderline Intellectual Functioning and Substance Use Disorder and Their Confidants: A Qualitative Study

**DOI:** 10.1080/19315864.2025.2459418

**Published:** 2025-01-31

**Authors:** Lotte C. F. Gosens, Robert Didden, Jannet M. de Jonge, Arnt F. A. Schellekens, Joanneke E. L. VanDerNagel, Roy Otten, Evelien A. P. Poelen

**Affiliations:** a Research & Development, Pluryn; b Behavioural Science Institute, Radboud University; c Trajectum; d ACHIEVE, Centre of Applied Research, Faculty of Health, Amsterdam University of Applied Sciences; e Department of Psychiatry, Radboudumc; f Nijmegen Institute for Scientist-Practitioners in Addiction, Radboud University; g Tactus, Centre for Addiction and Intellectual Disability (CAID); h Department of Human Media Interactions, University of Twente; i Aveleijn

**Keywords:** Motivational interviewing, cognitive behavioral therapy, substance use disorder, intellectual disabilities, qualitative study

## Abstract

**Introduction:**

Take it Personal!+ is a treatment program for individuals with Mild Intellectual Disability or Borderline Intellectual Functioning (MID-BIF) and Substance Use Disorder (SUD). It is supported by a mobile health application (mHealth), and researchers found it can reduce Substance Use (SU). Aims: This study aimed to explore the usability of the treatment program in as experienced by clients and their confidants.

**Methods:**

We conducted post-treatment, semi-structured interviews with clients (*n* = 8) and their confidants (*n* = 8). We coded transcripts according to thematic analysis and using inductive and deductive methods. Subsequently, we analyzed connections between the codes and grouped them into themes using axial coding.

**Results:**

Overall clients and confidants experienced the treatment program as usable, and most mentioned the program helped to reduce SU. The clients and confidants reported the presence of a confidant was helpful. Some clients and confidants reported the mHealth application was helpful. Components that were perceived as effective were self-control skills, daily registration exercise and discussing quantity of SU non-judgingly. Perceived impeding factors were video calling and a non-supportive network.

**Conclusion:**

This study shows that Take it Personal!+ is an useable treatment program for individuals with MID-BIF and SUD, that helps to decrease their self-reported SU. Nevertheless, there is room for improvement for further adapting the treatment, which will be discussed.

Individuals with Mild Intellectual Disability or Borderline Intellectual Functioning (MID-BIF; IQ range 50–85) have impairments in intellectual and adaptive functioning (i.e., conceptual, social and practical skills). These individuals frequently report comorbid mental health problems and experience difficulties in participation in society (American Psychiatric Association, 2022). They are at higher risk to develop Substance Use Disorder (SUD) and are overrepresented in addiction care (Didden et al., 2020). Approximately 30–40% of individuals in addiction care have a MID-BIF (Braatveit et al., 2018; Didden et al., 2020; Van Duijvenbode & VanDerNagel, 2019). However, general SUD treatment is often not adjusted to the needs of individuals with MID-BIF. For example, studies show access barriers to adequate SUD treatment and high drop-out rates in individuals MID-BIF in addiction settings (Krahn et al., 2006; Slayter, 2010; Van Duijvenbode & VanDerNagel, 2019).

Motivational Interviewing (MI) and Cognitive Behavioral Therapy (CBT) are widely used, effective treatment methods for SUD (Davis et al., 2015; Naar & Safren, 2017; Riper et al., 2014; Smedslund et al., 2011). Moreover, they are increasingly used in clinical practice for individuals with MID-BIF (Van Duijvenbode & VanDerNagel, 2019). While evidence for effectiveness in the general population is abundant (Davis et al., 2015; Naar & Safren, 2017; Riper et al., 2014), evidence for effectiveness in individuals with MID-BIF is limited (Didden et al., 2020; Van Duijvenbode & VanDerNagel, 2019). Take it Personal!+ is a personality-targeted, MI and CBT program adapted for individuals with MID-BIF and SUD (Gosens et al., 2021). A recent study using a multiple baseline across participants design showed that Take it Personal!+ program leads to a decrease in SU (Gosens et al., 2023). The most important adaptations in this treatment program included: 1) simplified communication, 2) a design of two sessions a week, which ensures repetition, 3) the presence of a confidant once a week, 4) a flexible treatment duration and 5) a supported Mobile Health (mHealth) application (for more information see Gosens et al., 2021).

Although the outcomes of treatment with Take it Personal!+ were promising (Gosens et al., 2023), additional, experiential information would be helpful to evaluate and improve the program. Therefore, the aim of this study was to explore the usability of Take it Personal!+ based on the experiences of clients and their confidants.

## MATERIALS AND METHODS

### Take it Personal!+

Take it Personal!+, supported by an mHealth application, is a personality-targeted MI-CBT SUD treatment, which aims to decrease SU in individuals with MID-BIF (Gosens et al., 2021). The treatment program is adapted to the specific personality profile (i.e., negative thinking, anxiety sensitivity, impulsivity and sensation seeking) of the client (Poelen et al., 2017). Based on the content and design, Take it Personal!+ usually lasts 11 weeks, but the duration can be adjusted based on the needs of the client. Take it Personal!+ consists of two, 45-min sessions a week and a confidant is present in one of these sessions. The confidant is chosen by the client from either their social network or professional-care network. The design of two sessions a week and the presence of the confidant ensures repetition of content and supports the generalization of learned skills to daily life, which is especially important in individuals with MID-BIF. The mHealth application supports the treatment and facilitates generalization of skills to daily life.

Take it Personal!+ consists of eight key components (see Table 1): 1) motivating to change SU, 2) psycho-education concerning the personality profile, 3) making a change plan and setting goals, 4) experiencing the personality profile and associated signals of problematic behavior, 5) functional analysis of SU, 6) increasing self-control skills, 7) behavioral and cognitive coping skills training and 8) relapse prevention. These key components are integrated into the curriculum of the treatment, and also incorporated in a mHealth application: TiP!. In TiP!, clients are able to do exercises, set goals, set future wishes, use the help!-button (i.e., receive personalized feedback or contact someone), consult key information (i.e., visible and audible), and consult their relapse prevention plan. Clients win accessories for their avatar and points for personal rewards by doing exercises and working on goals.

**Table 1.** Key components of the Take it Personal!+ treatment program.

	Description	Example
Week 1–2	Motivating to change SU	Focus on MI Exercises (i.e., “Identify problems,” “Cons of SU and pros of changing SU”)
	Psycho-education concerning the personality profile	Tailoring a game to the personality profile, and discussing it afterwards
	Making a change plan and setting goals	Mapping future wishes Making a change plan Setting specific goals (weekly goals are set from week 2 onwards)
Week 3–9	Experiencing personality profile and associated signals of problematic behavior	Doing a personality-specific game to recognize signals
	Functional analysis of SU	Making a functional analysis during the sessions (i.e., with use of exercises)
	Increasing self-control skills	Different self-control skills are discussed and learned through exercises (e.g., seeking distraction and thinking differently)
	Behavioral and cognitive coping skills training	Practicing skills through exercises (e.g., saying “no” in difficult situations)
Week 10–11	Relapse prevention	Making a relapse prevention plan

SU = Substance Use; MI = Motivational Interviewing.

In addition, clients make a daily registration exercise. This exercise, discussed during sessions, allows for identification of their quantity of SU and risk situations for SU, and to practice self-control skills. In this study, we used the mHealth application Ethica (EthicaData, 2019) for the daily registration exercise. Clients answered questions daily, to assess frequency and quantity of the primary SU, frequency of other SU, positive and negative affect, and a client-tailored question. For more information about the content of the treatment program, the mHealth application, and the adjustments to the needs of individuals with MID-BIF, see Gosens et al. (2021).

### Participants and Procedure

We conducted this qualitative study in two health care organizations for people with MID-BIF in the Netherlands. We invited 12 clients and their confidents from effectiveness study of Take it Personal!+ (Gosens et al., 2023) to participate. Of the 12 clients in the effectiveness study, three clients did not provide informed consent for the current qualitative study and one client was not able to participate due to clinical SUD treatment at the time of study. This resulted in eight clients and their confidants in this study. Four of the eight clients completed the treatment program with treatment durations between 5 and 11 months. The other four clients did not complete the treatment program. One client (P2) dropped out after eight sessions. The client reported low motivation and environmental factors that needed to change first. Two clients (P5, P10) were satisfied with the achieved results before finishing the full treatment and started a full-time job. One client (P3) was satisfied with the achieved results before completing the program and we referred the client to another mental health treatment. The treatment duration of these four clients lasted between 2 months and one year. The characteristics of the eight clients and their confidants are shown in Table 2.

**Table 2.** Participants’ characteristics (*N* = 8).

Characteristics	*N*
Age in years	18–35 (*m* = 24)
Gender	
Male	6
Substance Use Disorder (DSM-5)	
Moderate	2
Severe	6
Alcohol	2
Cannabis	5
Other drugs	1
Co-morbidity	
Attention Deficit Hyperactivity Disorder	3
Autism Spectrum Disorder	3
Posttraumatic Stress Disorder	2
Oppositional Defiant Disorder	1
Antisocial Personality Disorder	1
Reactive Attachment Disorder	1
Type of Confidant	
Professional caregiver	5
Mother	1
Brother	1
Girlfriend	1

Clients provided informed consent for the interview and audio recording at the start of treatment. At post-treatment, the first or last author contacted the client to schedule the interview and to receive the confidant’s contact details. If a client had more than one confidant, we asked the client to choose which confidant would be interviewed (i.e., mostly the confidant who was present most often during the sessions). The confidant received verbal and written information about the study and had a week to sign the informed consent form to participate in the interview and audio recording.

The first or last author, accompanied by an undergraduate research assistant, conducted post-treatment interviews. We had no prior relationship with the clients or confidants. We interviewed clients and their confidants separately unless the client preferred to be interviewed together with their confidant (*n* = 2). We conducted the interviews between November 2019 and November 2021. Initially, the interviews were face-to-face, but from February 2020 onwards they were held via video call, due to the COVID-19 pandemic. The length of the interviews varied from 30 min to 71 min, with a mean of 45 min. The Ethics Committee of the Faculty of Social Sciences of the Radboud University approved the study (ECSW-2019-033).

### Data Collection

To explore the experiences of the clients and their confidants, we developed two separate semi-structured interview guides each consisting of five topics: 1) treatment in general, 2) perceived effectiveness, 3) role of the confidant, 4) mHealth application TiP! and 5) maintaining learned skills in future. In addition, among clients who did not complete the treatment program, we explored reasons for drop-out. We also interviewed all confidants about the involvement of the social and professional network of the client. We explored each theme through several open-ended questions and questions were prompted to expand the responses.

### Data Analysis

We conducted and analyzed interviews in Dutch. We translated quotes into English for the manuscript. Two undergraduate research assistants audio-recorded, transcribed verbatim, and pseudo-anonymized (e.g., names of participants were replaced by numbers) all interviews. After additional probing, we imported the transcripts into the program ATLAS.ti to be coded according to thematic analysis (Braun & Clarke, 2006). First, the first author and a non-author separately coded the first transcript line-by-line using inductive and deductive methods (Corbin & Strauss, 2015). The first and last author then discussed the two coding lists with two non-authors and we agreed on a new coding list. Based on the new coding list, the first author coded all the transcripts line-by-line. After coding all transcripts, the first, second, and last author analyzed connections between the codes and we grouped into themes using axial coding (Corbin & Strauss, 2015). We reported results using the Standards for Reporting Qualitative Research (SRQR) (O’Brien et al., 2014).

## RESULTS

We identified the following themes: 1) general evaluation of Take it Personal!+, 2) evaluation of the adaptations to the needs of individuals with MID-BIF, 3) perceived treatment effectiveness, 4) perceived effective components and 5) perceived non-effective components. Within theme 2, we also identified four subthemes (i.e., two sessions a week, presence of a confidant, treatment duration and mHealth application TiP!). The themes and subthemes are described in the following paragraphs.

### General Evaluation of Take it Personal!+

Clients experienced the treatment as usable and informative. However, they also found it tough to be confronted with their SU. The clients mentioned some specific aspects of the treatment program were helpful. All clients had a therapist that was not previously involved in their care. The clients found this helpful, because they experienced no stigma and were thereby able to be honest and more open. One client said:

*“I did not know the therapist and she did not know me so then you already have a new start you know without stigma or something you know, that makes it easier for me to be open and honest, because the group I was in first if I was open about that [SU] it had immediately consequences.” (P3, non-completer).*

Also, all but one of the confidants mentioned it was better that the therapist had not been involved at an earlier stage in the clients’ care. One confidant said:

*“The therapist has also ensured that he [P2] always filled in everything honestly. The therapist just said very clear like I want to help you, but whether you take 4 joints, 5 or 6, I do not give a … I want to help you if you want to stop. And that message got him and I am sure that if there had been someone there who he already knew or who knew about his substance use or who had already expressed an opinion about it, he would not have picked up like that. Then he would have been much more closed. So for [P2] this was nice.” (Confidant P2, brother).*

All clients were content that Take it Personal!+ was an individual treatment and not a group treatment, because otherwise they would not have participated. They were reluctant to share their situation and thoughts with a group. One client said:

*“Then I am like I do not know them all and I do not want to share it with them just like that. I also do not know if these persons can be trusted that the person really keeps his mouth shut about that, you understand?” (P1, completer).*

One confidant mentioned that the therapist added treatment booster sessions after a couple of weeks. The client and confidant perceived these sessions as helpful and the confidant recommended to include this in the treatment program. The confident said:

*“What we are adding now is another booster session so I would definitely like to add that and maybe even more, more booster sessions. Yes it has to stop at some point, but I think that two booster sessions would be nice.” (Confidant P8, professional caregiver).*

### Evaluation of the Adaptations to the Needs of Individuals with MID-BIF

Most confidants mentioned that the treatment program met the needs of the clients. They specifically mentioned drawing and/or writing down the information, asking control questions, making information as simple as possible, repetition of content, no judgment and having the session at a place and time that was chosen or familiar by the client were important and helpful. One confidant reported:

*“A client with a mild intellectual disability just say that so there is a lot of repetition in it and a lot of uhm what you do you call that you just step back into something neutral without judgement because it turned out to be quite difficult for her to keep going and to keep to agreements [show up at sessions], that has cost us a lot of energy to try to keep that and I did have the idea that when she was doing that [show up at session] she was really more aware of her smoking behavior.” (P3, confidant, professional caregiver).*

One confidant mentioned that the therapist sometimes used words that were too difficult and that she could have used more simple words. She said:

*“I must say that the first few times I sometimes thought ooh that language used for our youngster that I thought ooh there are sometimes quite difficult words used. And our youngsters are not always those who do not always say that they do not understand or but sometimes I thought maybe that could be formulated differently.” (P4, confidant, professional caregiver).*

#### Two Sessions a Week

Four clients (P10, P14, P11, P16) mentioned having two treatment sessions a week was helpful. This way, the treatment was better incorporated in their daily life. One client said:

*“The timing was perfect so because if I uh from Thursday, Friday is towards the weekend, so you know what I mean. Anything can happen on the weekend. And when I come back on Monday, on the weekend I almost always think like eh you do not want to smoke on the weekend, because you have this on Monday, you have to say then Monday, you know, I smoked on the weekend. That was some of the things I put on my mind, you know. To make myself a little how do you say, not to smoke for a long time.” (P8, completer).*

The other four clients (P1, P3, P7, P4) mentioned having two sessions a week was not doable for them. Two clients mentioned they needed more time between two sessions to process what they have learned and to work on their goals. One client had a job and a frequency of two treatment sessions a week was not feasible. For one client the frequency of two sessions was too burdensome. One client reported:

*“I think now that I also work 5 days a week and I am busy. Actually I would not find it feasible to meet two times a week. So actually I just liked that [confidant] could be present and thereby one session. Because otherwise I think it would be too heavy for me.” (P4, completer*).

Two confidants mentioned two sessions a week was too burdensome for their client. One confidant was enthusiastic about the frequency and mentioned that it is important for the target group to have a lot of repetition. She reported:

*“A lot of things happens in a week and that [twice a week] also keeps it from weakening. At addiction care, for example, you were there once a week and then they had already forgotten what had been agreed. So keep coming backing with it [twice a week], in combination with the app, which is also used every day.” (Confidant P8, professional caregiver).*

#### Presence of a Confidant

Five clients chose a confidant from their professional caregivers at their treatment center. Clients were positive about the presence of the confidant and found this helpful. Also, the confidants were positive about their attendance. This enabled them to support the client outside the sessions. One confidant reported it was helpful for a trusting relationship between the therapist and client. Three of the five clients had one and the same confidant each session, which was feasible according to the confidants and this was helpful for the clients. One client and her confidant mentioned they preferred to have the same confidant each session, but if this was not possible, they preferred the attendance of another professional instead of no confidant at the session. One confidant reported:

*“If I would be the confidant of a new client, I would definitely choose to be present in each session, it would be actually a must for me so if the treatment will start at a day that I am not able to be present than the other professional need to be present. I really found this important. I also think that the great thing of a confidant during the sessions is that you can also come back to it in your care and that feels different if you were there than when you quickly read it back. I think that is quite a difference.” (Confidant P4, professional caregiver).*

Two clients had multiple confidants. The professional caregiver who was working that day joined the session. One of the clients mentioned that it was not helpful to have multiple confidants; he would prefer to have one or a maximum of two confidants. The other client was convinced he should work on his goals on his own. However, he also mentioned that sometimes it would have been more helpful if he had two confidants at most. The confidants of these clients did not see any problems in having more than one confidant and also mentioned that one confidant was not feasible. One client and his confidant reported:

*“I have experienced that three confidants were a bit too much, then it all get a bit mixed up, I found it a waste for the treatment. Mainly because the one did not know what the other had said and then it get mixed up. That was the only thing that bothered me a bit, but for the rest I also liked that there was someone there.” (P10, non-completer).*

*“I see that colleague who was also a confidant often so we were able to coordinate it well uhm and I think it is sometimes nice to switch things up a bit, especially because we are both very different so maybe we have a different view on things.” (Confidant P10, professional caregiver).*

Three clients had chosen a confidant from their social network and were also positive about their confidant. They experienced that the confidant got to know them better during the treatment, which strengthened their relationship in daily life. One client also mentioned he was glad that he did not have to tell things twice, because his girlfriend attended the session. One client said:

*“My brother also learned a lot of things about me, say, through what you thought, what the therapist asked you. My brother did not know things about me, that he does know now. In my opinion if people participate in Take it Personal!+ they should choose a family member, success guaranteed. It is only nice, for me he is still my confidant, and he will always be I think” (P2, non-completer).*

Two of the three confidants from the social network found it helpful to be the confidant during Take it Personal!+. In the beginning, one confidant had doubts if it would be helpful to attend as a mother, but she was glad that she did. Another confidant mentioned that the relationship with his brother improved, because he learned more about him. The last confidant mentioned that it was not necessary that she attended, because her boyfriend would tell her by himself what he had discussed. One confidant said:

*“I also learned more about him. So in that respect it was good too, for ourselves, uh for our relationship” (Confidant P2, brother).*

#### Treatment Duration

All clients and confidants were satisfied with the treatment duration. They mentioned that it was helpful that the treatment duration was personalized to the needs of the client. One confidant reported:

*“I think that it is nice for her that the time was just taken because there is just so much going on in her life that if there would also be some kind of time pressure behind it then she might have non-completer earlier and then it would have had little effect so it was nice that she [therapist] was so good and calmy taking her time” (Confidant P3, professional caregiver).*

#### mHealth Application TiP!

Some of the clients mentioned that the mHealth application TiP! was helpful for them and easy to use. Some mentioned that they liked that they could choose an avatar, doing exercises, reading information, having rewards and were able seeking for help. Some clients liked that they could keep using TiP! after they finished with Take it Personal!+. One client said:

*“I just liked that you could also put uhm in the app yourself, so I could put my own goals in the app.” (P2, non-completer).*

One client mentioned that the mHealth application TiP! was not helpful for him. He liked the rewards which motivated him to do exercises and work on goals, though it was not helpful for him. He said:

*“For example, if she [therapist] had that app with all those points, because she already discusses what is in that app anyway. And yes it is actually constant repetition*, *also. And for some it works because they need constantly repeat, repeat, repeat, but for me it just helps more that someone says to me verbally instead of such an app. You forget such an app again and verbally yes you cannot ignore that, it is the person you have to speak to, yes.” (P7, completer).*

Another client mentioned that it was a good mHealth application, but he wished he had used it more often. The mHealth application was less helpful for him, because he forgot to use it, as it did not send notifications. He said:

*“It was a good app, but if only I had, you know, filled in more or looked at it more. I mean it was always there, ‘ … .’ with this one [TiP!] I did not get a message and I am not really someone who is literally on their phone 24/7 or on social media all the time or whatever, you know and uh to me is just literal, if someone needs me I check my phone or whatever, Or if I want to know something, I just type on google.” (P8, completer).*

Some confidants mentioned that using the mHealth application in daily life led to increased motivation and awareness of clients’ treatment goal in daily life. Some confidants mentioned that clients could have used the mHealth application more often as less frequent use limited the mHealth’s contribution to treatment effectiveness. They recommended confidants to encourage the client to use the mHealth application and to build in notifications. One confidant reported:

*“In itself it is a nice app, he [P10] also knew exactly how it worked so I think he actually used it uhm it is a good way to make someone involved in their own process uhm I did not think it was too childish either, that is often still a pitfall with this target group, so no, I was positive.” (Confidant P10, professional caregiver).*

### Perceived Treatment Effectiveness

With regard to perceived treatment effects on SU, four clients mentioned that they had quit SU and the other four clients mentioned that they had cut down their SU. Clients believed the Take it Personal!+ treatment helped them. One client reported:

*“I think without her [therapist] I would still be addicted I think. Or not addicted but still using and then uh I have now uh this will be my fifth month clean.” (P5, non-completer).*

The confidants reported the same results as the clients. One confidant reported:

*“Without Take it Personal!+ ‘ … ’ he [P2]could not have done it like that. If I had said to him, without Take it Personal!+, you have to put less cannabis in your joints, he would not have done that. So in that, it is a direct result of Take it Personal!+ in this case. From the sessions that we have had and that we have been able to discuss it like this and that he has been able to practice with it and make mistakes that yeah, without it [Take it Personal!+] he would never have achieved that little sub-goal. And neither do the rest. No he could not have done that on his own. So praise in that regard, but unfortunately he did not achieve the desired treatment goal.” (Confidant P2, brother).*

Further, clients mentioned other changes in their life. Some clients reported they were less influenceable, had more self-confidence, and a higher self-image. One client said:

*“For example, ‘take something too, take something too’ then I already took it. But if they ask that now, I will immediately leave the situation, go home or leave. At least out of the situation and that did help me” (P5, non-completer).*

One client mentioned that friendships became stronger and another client

mentioned she more often engaged in social contacts. She said:

*“I have become socially higher actually, because like yes there is another girl here [building] with whom I just have contact with. I occasionally eat with and just smoke [cigarettes] with her. Look, that social has suddenly gone up and I actually did not have that social either. I was just a girl who was sitting upstairs smoking weed and doing my thing and not really looking at people. ‘ … ..’ I was just socially awkward, but since I really quit [with cannabis] yes that just helped socially up actually. So I am happy with that, because otherwise I would still have been sitting here in my room and I would not have done a damn thing. So yes and my house is still in order, that was not the case before. So yeah I am just proud.” (P4, completer).*

Other changes clients mentioned were being more able to talk about SU, including being open and honest and asking for help. One client mentioned he now realized that he does not have to do things all by himself. One client reported:

*“I do not hide myself anymore and yes I just dare to stand there since I did Take it Personal!+ because I have been walking alone for a long time with my addiction, with smoking weed. I thought I cannot tell anyone because uhm then I will disappoint people and you know this and that and now I am just very open about it and especially if I can help other people you know. I am not someone who uhm will lie about that like no I do not do it [SU] while I do, you understand. And yes I am very grateful for that.” (P3, non-completer).*

Some clients mentioned they felt physically better. One client said:

*“I exercise again 3.5 hours a day, great so I can do many more things again. I do not sweat so much anymore, I sleep better yes I do not have a headache anymore. I do not constantly need to drink. If you drink every day you constantly need it too. Yes, there are many advantages.” (P7, completer).*

The confidants mentioned similar changes in clients, including: less influenceable, a more positive self-image, improved self-care and housekeeping, and being more able to talk about feelings. Some confidants also reported that the clients talked more openly about SU with their network and confidant. One confidant said:

*“She is more open about it, just to say, she also say right away oh I slipped up, it also nice that this terminology sticks with her, and she can now really talk about that.” (Confidant P3, professional caregiver).*

### Perceived Effective Components

There was substantial variation in the clients’ perception of effective components. Some clients mentioned the self-control skills (i.e., indicating and discussing, taking distance, seeking distraction, alternatives, applause and thinking differently) as most helpful. Some clients mentioned that the daily registration exercise, combined with the intensity of two sessions a week, was most helpful for them because treatment became part of their daily life. For some clients, learning how to deal with leisure time was most helpful. Finally, for some clients, discussing non-judgingly the quantity of SU and the moments of SU during sessions was most helpful. One client reported:

*“Yes those questions [daily registration exercise], then you will be reminded every day that you should not use substances. ‘ … ..’ I liked it yes, because that reminds you that you have to stick to the flag and uhm and what you did that day and uh how much and yes. Who were you with and yes. I liked it. It was helpful, it made me think of it [treatment goal].” (P5, non-completer).*

Confidants also differed in the components that were the most helpful for the clients: discussing SU in a normal manner, being honest about SU, daily registration exercise, setting (small) treatment goals and writing them down each week, obtaining success experiences, psycho-education regarding SUD and personality profile, self-control skills, two sessions a week, mind-set in relapse, dealing with leisure time and repetition of content. Two confidants mentioned that it was difficult for the clients to show up each session. A confidant also reported that continued treatment even after multiple client absences was helpful, otherwise results would not have been achieved. One confidant reported:

*“And so there was every week he got that structure and especially the app [daily registration exercise] et cetera that secretly helped him in his subconscious because that made him busy with it every time and he was reminded every day of hey I should not drink too much and what did I drink and what not and I think that makes it clear to him. Because of that he had to do things uhm he got confronted with his alcohol consumption every day.” (Confidant P1, professional caregiver).*

### Perceived Non-Effective Components and Impeding Factors

We also explored the non-effective components of Take it Personal!+. Clients and confidants differed in components that were least helpful in obtaining treatment goals. Two clients and their confidant mentioned that they did not use the relapse prevention plan. One confidant mentioned that filling in the daily registration exercise was least helpful for the client. She said:

“*I think the least helpful were filling in the things [daily registration exercise], because every time she [P3] had to say no, I did not succeed.” (Confidant P3, professional caregiver).*

In addition, we explored impeding factors in obtaining treatment goals. One client and another confidant experienced video calling during the COVID-19 pandemic as not being helpful. One confidant said:

*“I was video calling with [therapist] then it was a bit disturbing now and then that video calling, because you do not always get everything, or [therapist] did not hear it well or one of us did not hear it well or, yes. Then notice if you say that you are personally sitting in front of someone that is just a little bit more, then it is easier to grab a sheet of paper again to draw something or to write something down to clarify it a bit and that is lacking then” (Confidant P3, professional caregiver).*

Other clients mentioned that non-supportive relationships/network and low self-efficacy were not helpful in obtaining treatment goals. One client reported:

*“When I entered the relationship, yes the relationship is over now, I was influenced in a negative way uhm if you are going to challenge me like ‘oh no, you cannot do this or that,’ then I will go do it right away you know and if I then ask you would you please go outside with me or just go into the woods, just to clear my head. And you put me down every time and say I am weak, that kind of thingss then it is easier for me to reach for drugs.” (P3, non-completer).*

Finally, confidants also mentioned having a non-supportive relationships/network was not helpful. One confidant reported:

*Her entire network is just occasionally problematic, which creates a lot of tension that makes her just really go back to smoking weed again. (Confidant P3, professional caregiver).*

## DISCUSSION

The aim of this study was to explore the usability of Take it Personal!+, a personality-targeted SUD treatment for individuals with MID-BIF and SUD (Gosens et al., 2021), based on experiences of clients and their confidants. This study showed that clients and confidants generally experience Take it Personal!+ to be usable and helpful. In clinical practice, SUD treatment is commonly provided in a group. Though earlier studies in individuals albeit without MID-BIF showed no differences in treatment outcomes between group and individual treatment (Epstein et al., 2018; Weiss et al., 2004), a recent study showed small effects of group treatment in abstinence rates compared to individual treatment (Lo Coco et al., 2019). However, research in individuals with MID-BIF comparing group and individual SUD treatment is lacking. Interestingly, clients were content about the fact that Take it Personal!+ was an individual treatment instead of a group treatment.

Concerning the adaptations of the treatment program to the needs of people with MID-BIF, this study showed that half of clients perceived two treatment sessions a week as helpful. The other half mentioned two sessions a week was not doable for them. Reasons for this varied among clients. Some clients needed more time between two sessions to process the session information and work on their goals, while another client had too little time for two weekly sessions due to a job. This underlines the heterogeneity of the target group and the need of attuning the treatment program to the characteristics and circumstances of the individual client.

Another important adaptation of Take it Personal!+ for individuals with MID-BIF is the presence of a confidant. Both clients and confidants found the role of the confidant supportive during Take it Personal!+. Only one confidant mentioned it was not necessary that she attended the sessions. This is in line with research, showing the presence of a caregiver during depression treatment is valuable for individuals with MID-BIF, because the caregivers were able to support the individuals implementing skills in daily life and after treatment (Hartley et al., 2015). In addition, clients and confidants mentioned their relationship improved. However, it is unknown to what extent the presence of a confidant enhances treatment effects. In our study, clients perceived the presence of a confidant as less effective if there were more than two confidants, because each confidant was not aware of the treatment process. Further research is necessary to examine the added value of confidants in the treatment for people with MID-BIF.

The clients and confidants perceived the flexible treatment duration as helpful. One confidant recommended to extend the treatment duration with two booster sessions (i.e., content of treatment can be repeated, personalized to the client) after a couple of weeks. Most individuals with MID-BIF experience difficulties in memory, particularly individuals with SUD (Willner & Lindsay, 2016), which supports the recommendation to add booster sessions after a couple of weeks. In addition, this recommendation is also in line with SU research in individuals without MID-BIF, which shows a positive effect of booster sessions in treatment outcome (Zlotnick et al., 2009)

Using an mHealth application during SUD treatment in individuals with MID-BIF was feasible, which is in line with other studies on digital support in CBT (Vereenooghe et al., 2016). However, the perceived effectiveness – in terms of obtaining treatment goals – of the mHealth application differed between the clients. This study provides a first indication that clients have to be reminded to make exercises, read information, read their goals by notifications send by the mHealth application, and the support of a confidant using an mHealth application might be important to support SUD treatment in this target group. In line with other studies (e.g., Raspa et al., 2018), we recommend further study of the specific design features (e.g., notifications) of mHealth for individuals with MID-BIG. Additionally, the effectiveness of the mHealth application TiP! in obtaining treatment goals is unknown and we recommended to test the mHealth application using an experimental design comparing Take it Personal!+ with mHealth application TiP! with Take it Personal!+ without mHealth application TiP!.

Regarding perceived treatment effectiveness, clients believed Take it Personal!+ helped to reduce their SU, which is in line with the results from the quantitative intervention study (Gosens et al., 2023). One of the effective components in obtaining treatment goals was the daily registration exercise, because in this way the treatment was part of their daily life. This is important because individuals with MID-BIF experience problems in generalizing learned skills to daily life (Willner & Lindsay, 2016). One client and another confidant experienced therapy delivered through video calling, necessitated by the COVID-19 pandemic, as an impeding factor as they preferred face-to-face treatment sessions. In clinical practice of treatment of people with MID-BIF, video calling is increasingly used, though studies on the usability of video calling in individuals with MID-BIF are still limited. Two studies showed beneficial effects as well as limitations of video calling in individuals with MID-BIF (Oudshoorn et al., 2023; Rawlings et al., 2021). Based on these studies and the experiences in the present study, we recommend further research to determine if video calling has added value when providing SUD treatment to individuals with MID-BIF.

In addition, having a non-supportive network of substance using peers and relatives was mentioned by several clients and confidants as an impeding factor in obtaining treatment goals. They did not experience (emotional) support from people in their network who also used substances. This is in line with research showing a higher frequency of SU in individuals with a network of users compared to individuals with a social network of abstainers in public schools (Ennett et al., 2006). This underlines the urgency of discussing the clients’ network during treatment to obtain a supportive network (i.e., network who supports the client in obtaining treatment goals).

One of the major strengths of this study are the unique analyses of perspectives on Take it Personal!+. First, we interviewed clients as well as confidants. Second, we interviewed both clients who completed the treatment as well as non-completers. And last, we interviewed both confidants from clients’ professional network and from social networks. Interestingly, we did not find many differences between the perspectives. One client and his confidant only differ in the fact of having more than one confidant. The client did not find it helpful to have multiple confidants, whereas the confidant did not see any problems in having more than one confidant.

Despite these promising and unique results, this study has some limitations. The study was conducted in only two health care organizations in the Netherlands with a small group of participants (*n* = 8). This limits the generalizability of our conclusions, and the conclusions need to be interpreted with caution. Second, we did not explore the perspectives of the therapists. Finally, a part of the interviews were conducted during a video call due to the COVID-19 pandemic, which may have influenced the interaction with the interviewer (e.g., non-verbal behavior is more difficult to see) as another study also mentioned (Husain et al., 2023).

In conclusion, Take it Personal!+ seems to be an usable treatment program in individuals with MID-BIF and SUD, which can be helpful in decreasing their SU. This study indicates that personalizing treatment with individuals with MID-BIF and SUD is important due to their heterogeneity. In clinical practice, therapists need to be aware of the heterogeneity and thus attune the treatment program to the characteristics and circumstances of the individual client.
